# Folate-targeting annonaceous acetogenins nanosuspensions: significantly enhanced antitumor efficacy in HeLa tumor-bearing mice

**DOI:** 10.1080/10717544.2018.1455761

**Published:** 2018-04-02

**Authors:** Haowen Li, Yijing Li, Hui Ao, Dongdong Bi, Meihua Han, Yifei Guo, Xiangtao Wang

**Affiliations:** Institute of Medicinal Plant Development, Chinese Academy of Medical Sciences & Peking Union Medical College, Beijing, P. R. China

**Keywords:** Annonaceous acetogenins, folate targeting, nanosuspensions, DSPE-PEG-FA, PEGylation, anti-tumor efficiency

## Abstract

Annonaceous acetogenins (ACGs) are one of the most active constituents isolated from Annona species with potent antitumor activity. However, the poor solubility and severe side effect greatly limit their use in clinic. In this study, folic acid (FA) modified annonaceous acetogenins nanosuspensions (FA-PEG-ACGs-NSps) had been successfully prepared using DSPE-PEG-FA and soybean lecithin (SPC) as stabilizers. The resultant FA-PEG-ACGs-NSps had a mean particle size of 119.7 nm, a zeta potential of –23.0 mV and a high drug payload of 49.68%. The obtained ACGs-NSps had a good stability in various physiological media, and showed sustained drug release. Compared to common ACGs nanoparticles (PEG-ACGs-NSps), FA-PEG-ACGs-NSps showed significantly enhanced *in vitro* cytotoxicity against folate receptor-positive HeLa cell lines (IC50, 0.483 μg/mL vs. 0.915 μg/mL, *p* < .05), which could be effectively reversed simply by pretreatment of free FA. *In vivo* experiments demonstrated that FA-PEG-ACGs-NSps brought more drug molecules into tumors and greatly improved the antitumor efficacy (TIR, 76.45% vs. 25.29%, *p* < .001). Therefore, DSPE-PEG-FA is considered as a proper stabilizer with active targeting effect for ACGs-NSps to reduce toxicity, enlarge the safe dosage range and apply in clinic for the treatment of folate-positive tumors. Therefore, FA-PEG-ACGs-NSps may be a prospective drug delivery system for ACGs to improve their therapeutic window and find application in clinic to treat FR over-expressed tumors.

## Introduction

Annonaceous acetogenins (ACGs) are one of the most active constituents isolated from Annona species (Annonaceae) (Rupprecht et al., 1990). ACGs are a group of C_35_ or C_37_ compounds bearing a terminal methyl-substituted α, β-unsaturated γ-lactone ring with 1, 2, or 3 tetrahydrofuran (THF) rings (Pan & Yu, 1997; Bermejo et al., 2005; Chen et al., 2011). Owing to their potential antitumor activity, ACGs have been widely concerned and have aroused extensive research in the past three decades (Pieme et al., 2014; Formagio et al., 2015; Bomfim et al., 2016). Annonaceous acetogenins and the single compounds separated from ACGs exhibited antitumor activity against various cancer cells, such as HeLa, HepG2, SMMC-7721, MCF-7, and MKN-45 cell lines (Chen et al., 2012). For example, squamocin, one of the most active components of ACGs, showed an IC50 value of 0.5 nΜ against MCF-7 cells, which was approximately 100 times more active than adriamycin (Yang et al., 2009). Bullatacin, another major component of ACGs, was 400 times as active as Taxol, confirmed by L1210 murine leukemia bearing mice (Ahammadsahib et al., 1993). Recently, three new constituents of ACGs extracted from the seeds of *Annona squamosa* demonstrated high potent cytotoxic activity against HepG2, H460, and BGC-803 with IC50 values of 0.43, 0.103, and 0.687 μg/mL, respectively (Chen et al., 2011; Sun et al., 2014; Sun et al., 2015). It is obviously that ACGs possessed stronger toxicity than other common antitumor drugs.

It was reported that ACGs could inhibit the mitochondrial respiratory chain complex I and nicotinamide NADH (nicotinamide adenine dinucleotide) oxidase (Degli et al., 1994; Tormo et al., 1999; Motoyama et al., 2002; Duval et al., 2006; Zafra-Polo et al., 2010), and then block the electron transport chain and reduce the production of ATP. This also explains why ACGs can kill multiple drug resistant (MDR) tumors for MDR pump requires the support of ATP (Tormo & Estornell, 2000).

However, the very poor solubility (lower than the lowest quantification limit of the analysis method) (Dang et al., 2012) greatly restricted their drug delivery and implementation of their *in vivo* antitumor efficacy. Nanotechnology has long been regarded to be one of the most effective strategies to solve this problem. Nanosuspensions (NSps) have high biocompatibility, long-time circulation, low toxicity and efficient drug loading efficiency, and then has attracted more and more attention by researchers working on targeted and sustained drug-release system (Jain, 2005; Aditya et al., 2015; Xu et al., 2017; Yang et al., 2017). In addition, intravenously administrated NSps can effectively accumulate in tumor tissue due to the enhanced permeability and retention (EPR) effect (Torchilin, 2007; Jain et al., 2008; Chen et al., 2011). In our previous research, we have prepared ACGs-NSps stabilized by PEG2000-PCL2000 or self-assembled cyclodextrins–SPC complex. The resultant ACGs-NSps showed a small size, good stability, stronger *in vitro* cytotoxicity and significantly improved *in vivo* antitumor efficacy than traditional ACGs oil solution (Aditya et al., 2015; Hong et al., 2016). Nowadays, folic acid (FA) has become the most widely used tumor targeting ligand with a high affinity to folate receptor (FR)-positive cells (Leamon & Low, 1991; Leamon & Reddy, 2004; Reddy et al., 2006). Folate receptor α (FRα), a glycosylphosphatidylinositol glycoprotein anchored to cell surface, is overexpressed in various epithelial malignant cancers, including ovarian, breast, and lung cancer (Bueno et al., 2001; Matherly & Goldman, 2003; Lu et al., 2005; Kalli et al., 2008; Kennedy & Low, 2011; Christoph et al., 2013). But FRα expression is limited in normal tissues (Weitman et al., 1992). Due to this special expression pattern, the folate-modified drug delivery systems carrier binding with FRα can be selectively transported into tumor cells via the endocytic process (Solanky et al., 2010) and simultaneously decrease drug toxicity to normal cells. In this study, DSPE-PEG2000-FA was used as a stabilizer to prepare ACGs-NSps and meantime to provide the resultant ACGs-NSps with active target ability to FR overexpressed tumor and long blood circulating time, aiming at good *in vivo* antitumor efficacy superior to the common ACGs-NSps.

## Materials and methods

### Materials

Annonaceous acetogenins were provided by Prof. Wenhua Huang from the Institute of Medicinal Plant Development (IMPLAD, batch number: 091, the composition was seen in Table S1); the DSPE-PEG-FA and DSPE-PEG were purchased from Shanghai ToYong Biotechnology Company Ltd (Shanghai, China). Soybean lecithin (SPC) was obtained from Guangzhou Hanfang Pharmaceutical Company Ltd (Guangzhou, China). The 3-(4,5-dimethylthiazol-2-yl)-2,5-diphenyltetrazolium bromide (MTT) was provided by Sigma-Aldrich Co. (St. Louis, MO). DiR was purchased from AAT Bioquest Inc. (Sunnyvale, CA). PTX injections were supplied by Beijing Union Pharm Ltd (Beijing, China). All the other reagents were of analytical grade or higher. The water used in the experiments was deionized.

### Animals and cell lines

KunMing (KM) mice and female Balb/c nude mice (6–8 weeks old, 20 ± 2 g) were purchased from the Vital River Laboratory Animal Technology Co, Ltd (Beijing, China). All animal experiments followed the principles of the Ethical and Regulatory Guidelines for Animal Experiments as defined by IMPLAD, Beijing, China. The ethics committee of IMPLAD granted ethical approval for this study. The animals were kept at 25 °C and a relative humidity of 70% ± 5% under 12 h light–dark cycle conditions for 1 week prior to the experiments. The HeLa (cervix carcinoma) and A549 (human lung carcinoma) were purchased from China infrastructure of cell line resource. The cells were both cultured in Dulbecco’s Modified Eagle’s Medium (Thermo Fisher Scientific, Waltham, MA) containing 10% fetal calf serum (Thermo Fisher Scientific, Waltham, MA), 100 U/mL penicillin and streptomycin (Gibco, St Louis, MO) at 37 °C with 5% CO_2_ (Sanyo, Osaka, Japan).

### Preparation of FA-PEG-ACGs-NSps

FA-PEG-ACGs-NSps were prepared using a precipitation-ultra sonication method as described in our previous study (Hong et al., 2016). Briefly, 6 mg of ACGs, 3 mg of DSPE-PEG-FA and 3 mg of SPC were co-dissolved in 0.5 mL of methanol, then the mixed solution was injected dropwise into 6 mL of deionized water at 28 °C ± 2 °C under ultra-sonication at 250 W for 10 min (Kun Shan Ultrasonic Instruments Co., Ltd, Kunshan, PR China), followed by evaporation of methanol under vacuum at 40 °C until no organic solvent remained. PEG-ACGs-NSps (without FA) were prepared via the same method using DSPE-PEG instead of DSPE-PEG-FA.

In order to trace the bio-distribution of ACGs NSps, DiR, a hydrophobic, near-infrared fluorescent dye, was incorporated into the hydrophobic cores of ACGs-NSps to visualize the *in vivo* biodistribution of ACGs. Preparation of DiR-loaded NSps was prepared according to the same procedure as described above, except for the incorporation of DiR in ACGs (ACGs:DiR =40:1, weight ratio) before dissolution in methanol.

### Characterization of ACGs-NSps

The average particle size, polydispersity index (PDI), and zeta potential of FA-PEG-ACGs-NSps and PEG-ACGs-NSps were measured by dynamic light scattering (Zetasizer Nano ZS; Malvern Instruments, Malvern, UK) at 25 °C. Each sample was measured in triplicate with 12 scans.

The morphology of FA-PEG-ACGs-NSps and PEG-ACGs-NSps was observed using a JEM-1400 transmission electron microscope (TEM; JEOL Ltd, Tokyo, Japan). One drop of the samples was placed onto a 300-mesh copper grid, air-dried, and negatively stained with 2% (w/v) uranyl acetate to observe under electron microscope.

Drug loading content (DLC) was determined by dissolving FA-PEG-ACGs-NSps in 9-fold volumes of acetonitrile to disintegrate the structure of NSps and release the encapsulated ACGs, and the mixture was then passed through a 0.22 μm syringe filter for high-performance liquid chromatography (HPLC) analysis. Drug loading content (DLC%, w/w) was calculated as follows:

(1) $$\text{DL}\%=W_{\text{drug}}/W\times \text{1}00\%$$

where *W*_drug_ is the mass of the drug in FA-PEG-ACGs-NSps and *W* is the weight of lyophilized powder of FA-PEG-ACGs-NSps containing both the drug and stabilizer.

### Chromatographic conditions for HPLC analysis of ACGs

The content of the ACGs was determined by HPLC using bullatacin as an indicator (a major component of ACGs). The HPLC system (DIONEX Ultimate 3000, Sunnyvale, CA) was equipped with an auto sampler and the chromatographic separation was performed using a Symmetry C18 column (4.6 mm × 250 mm, 5 μm, Waters, ‎Milford, MA) at 30 °C. The mobile phase was composed of acetonitrile and water (70/30, v/v). The flow rate was 1 mL/min. The detection wavelength was set at 210 nm (UV detector, DIONEX). For DiR analysis, the same HPLC system was used in combination with a fluorescence detector set at excitation wavelength =748 nm and emission wavelength =780 nm. The mobile phase was a mixture of acetonitrile and water (90:10, v/v) with a flow rate of 1.0 mL/min at 30 °C.

### Stability of ACGs-NSps in various physiological solution

FA-PEG-ACGs-NSps (1 mg/mL) and PEG-ACGs-NSps were mixed (1:1, v/v) with 1.8% NaCl, 10% glucose, and 2 × PBS (pH 7.4), respectively, and then incubated at 37 °C. At specific time intervals, a 1 mL mixture was taken out and analyzed for size change and particle distribution. Each experiment was performed in triplicate.

### Stability of ACGs-NSps in rat plasma

To study whether plasma components (including enzymes and serum albumin) could interact with FA-PEG-ACGs-NSps or PEG-ACGs-NSps and then induce aggregation, *in vitro* plasma stability research was conducted as follows: FA-PEG-ACGs-NSps (1 mg/mL) and PEG-ACGs-NSps (1 mg/mL) were mixed with rat plasma (1:4, v/v) respectively and incubated at 37 °C. At each time interval, 1 mL mixture was removed and analyzed for size change and particle distribution. Each experiment was performed in triplicate.

### *In vitro* drug release behavior

FA-PEG-ACGs/DiR-NSps (4 mL, 1 mg/mL) and PEG-ACGs/DiR-NSps (4 mL, 1 mg/mL) were placed in Float-A-Lyzer dialysis cassettes (molecular weight cutoff 20 kDa; Spectrum Labs, Rancho Dominguez, CA, USA), immersed into 2 L of PBS (pH 7.4, 0.1 mol/L), and incubated at 37 °C with stirring (100 rpm). At specific time intervals, 50 μL of the dialysate inside the cassettes was taken out, mixed with 450 μL of methanol for disintegration of nanoparticles and release of the encapsulated drug. Then, the mixture was filtered through a 0.22 μm syringe filter and then analyzed using HPLC analysis for the concentration of ACGs and DiR. Each time, the cassettes were replenished with 50 μL of fresh PBS solution. The release medium was replaced every 24 h. All assays were performed in triplicate.

### *In vitro* cytotoxicity assay

The *in vitro* cytotoxicity of FA-PEG-ACGs-NSps against FR-positive HeLa cells and FR-negative A549 cells was evaluated using MTT assay. One hundred and fifty microliters HeLa/A549 cells (8.0 × 10^3^ cells/well) were seeded in 96-well plates and incubated overnight in 5% CO_2_ at 37 °C. Then, the medium was removed and various concentrations of FA-PEG-ACGs-NSps, PEG-ACGs-NSps, the physical mixtures of FA and PEG-ACGs-NSps, the physical mixtures of FA and FA-PEG-ACGs-NSps and free ACGs solution (dissolved in DMSO, final concentration ≤0.1%) were added and incubated for 24 h. Then, the cells were treated with 20 μL MTT solution (5 mg/mL in PBS) and incubated for 4 h. Finally, the medium was replaced with 150 μL of DMSO, the maximum absorbance of DMSO solutions was detected at 570 nm using an ELISA plate reader (Biotek, Winooski, VT). The cell viability rate was measured as follows:

$$\text{Cell viability rate }\left(\%\right){\text{=OD}}_{\text{t}}\text{/}{\text{OD}}_{\text{c}}\times 100\%$$

where OD_t_ was the mean absorbance (OD) of tested group and OD_c_ was the mean OD of control group.

The IC50 value for each group was determined using GraphPad Prism Software, Version 5 (GraphPad Software, Inc., La Jolla, CA).

### *In vivo* antitumor activity study using HeLa tumor-bearing mice

Female Balb/c nude mice (20 ± 2 g) were inoculated subcutaneously with HeLa cells (1.0 × 10^6^ cells/mouse) in the right armpit. When the tumor size reached 100 mm^3^, the mice were randomly divided into four groups (eight mice per group) as follows: normal saline (negative control), PTX injection (positive control, 8 mg/kg, iv), FA-PEG-ACGs-NSps (0.4 mg/kg, iv), and PEG-ACGs-NSps (0.4 mg/kg, iv). All the groups were administered intravenously through the tail vein every other day for 2 weeks. The body weight and the tumor volume were measured throughout the experiment.

The tumor inhibition rate (TIR%) was calculated as follows:

$$\text{TIR}\%=\left(\text{1}-W_{\text{t}}/W_{\text{n}}\right)\times \text{1}00\%$$

where *W*_n_ is the average tumor weight of negative control group and *W*_t_ is the average tumor weight of the mice in the tested group.

### *In vivo* biodistribution using HeLa tumor-bearing mice

Female Balb/c nude mice (20 ± 2 g) were inoculated subcutaneously with HeLa cells (1.0 × 10^6^ cells/mouse) in the right armpit. When the tumor size reached 500 mm^3^, the mice were randomly divided into two groups (eight mice per group), one was intravenously administrated with FA-PEG-ACGs/DiR-NSps (0.4 mg/kg, iv), another was administrated with PEG-ACGs/DiR-NSps (0.4 mg/kg, iv). Twenty four hours later, the mice were sacrificed, and the tumor tissues and the major organs (heart, liver, spleen, lung, kidney) were excised and imaged using IVIS Living Image^®^ 4.4 (Caliper Life Sciences, Hopkinton, MA). Living Image software (version 4.2) was used for quantitative analysis.

### Statistical analysis

The statistical analysis was performed using the independent-samples *t*-test with IBM SPSS Statistics Software, Version 19 (IBM Corporation, Armonk, NY). *p* < .05 was considered statistically significant.

## Results and discussion

### Preparation and characterization of FA-ACGs-NSps

Stabilizer plays an important role in the preparation and the *in vivo* behavior of the resultant NSps. DSPE-PEG-FA was selected as the major stabilizer because its ‘FA’ moiety can provide the obtained ACGs-NSps with the ability of active targeting to ‘FA’ receptor on the surface of HeLa cells in tumor of model mice while its ‘PEG’ segment can provide ACGs-NSps with long blood circulation time after intravenous administration.

However, when DSPE-PEG2000 or DSPE-PEG2000-FA was used alone, the resultant ACGs-NSps were bigger in size (Table S2) and induced obvious hemolysis to red blood cells (Figure S2). If SPC was used in combination as a stabilizer, the hemolysis was reduced and the mean particle size of the resultant ACGs NSps was also decreased (Table S2 and Figure S2). So, it was determined that DSPE-PEG2000 or DSPE-PEG2000-FA was used together with SPC to prepare ACGs-NSps in the subsequent study. Meanwhile, different proportion of DSPE-PEG2000-FA/SPC was tried in order to obtain the smallest ACGs nanoparticles. As seen in Table S3, when DSPE-PEG-FA/SPC weight ratio was 1:1, FA-PEG-ACGs-NSps reached the smallest average diameter (119.7 nm) with presentable PDI value (0.20) and zeta potential (–23.0 mV). Since the small nanoparticles had advantage over larger ones in the tumor penetration and accumulation (Chen et al., 2011), ACGs/DSPE-PEG-FA/SPC (2:1:1, weight ratio) was selected as the optimal formulation in the preparation of FA-PEG-ACGs-NSps for the subsequent use. HPLC analysis that the DLC of FA-PEG-ACGs-NSps was 49.68%, was in good accordance with the theoretical DLC (50%).

**Figure 1.**
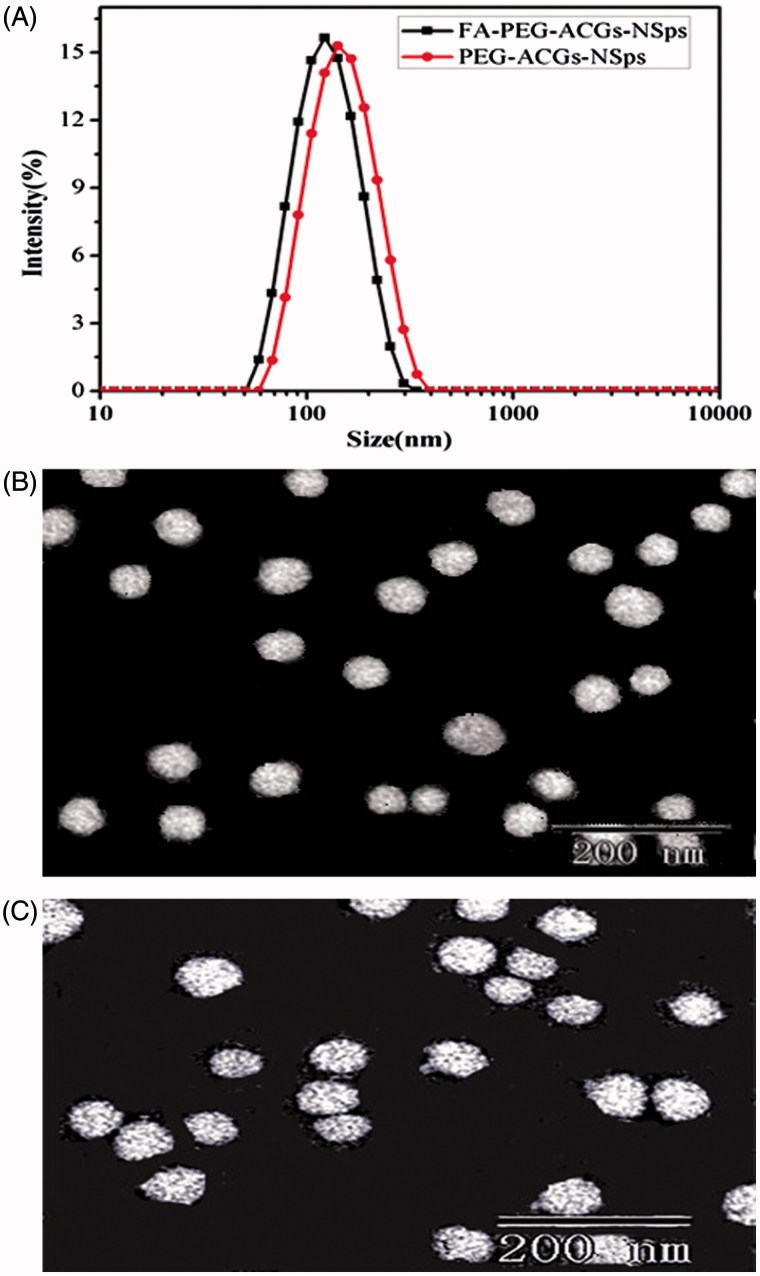
The particle size distribution and morphology of ACGs-NSps. (A) The particle size of FA-PEG-ACGs-NSps and PEG-ACGs-NSps measured by DLS. (B) TEM micrograph of FA-PEG-ACGs-NSps. (C) TEM micrograph of PEG-ACGs-NSps.

Correspondingly, ACGs/DSPE-PEGA/SPC (2:1:1, weight ratio) was employed for the preparation of PEG-ACGs-NSps. The resultant PEG-ACGs-NSps showed a little larger particle size (139.0 ± 1.9 nm) than FA-PEG-ACGs-NSps (Figure 1(A)) with a PDI value of 0.209 and a zeta potential of –30.1 mV. Transmission electron microscopy observation revealed that both FA-PEG-ACGs-NSps (Figure 1(B)) and PEG-ACGs-NSps (Figure 1(C)) were spherical, and uniform in shape. The size was in good agreement with that determined by dynamic light scattering.

Incorporation of DiR displayed not much effect on the preparation and the physical properties of ACGs-NSps. As seen in Table S4, the obtained FA-PEG-ACGs/DiR-NSps and PEG-ACGs/DiR-NSps showed mean diameter of 131.0 nm and 132.5 nm, respectively, with narrow particle size distribution (PDI values being 0.183 and 0.207, respectively) and a little increased zeta potential (–30.1 mV and –36.1 mV).

### Stability of ACGs-NSps in physiological media

**Figure 2.**
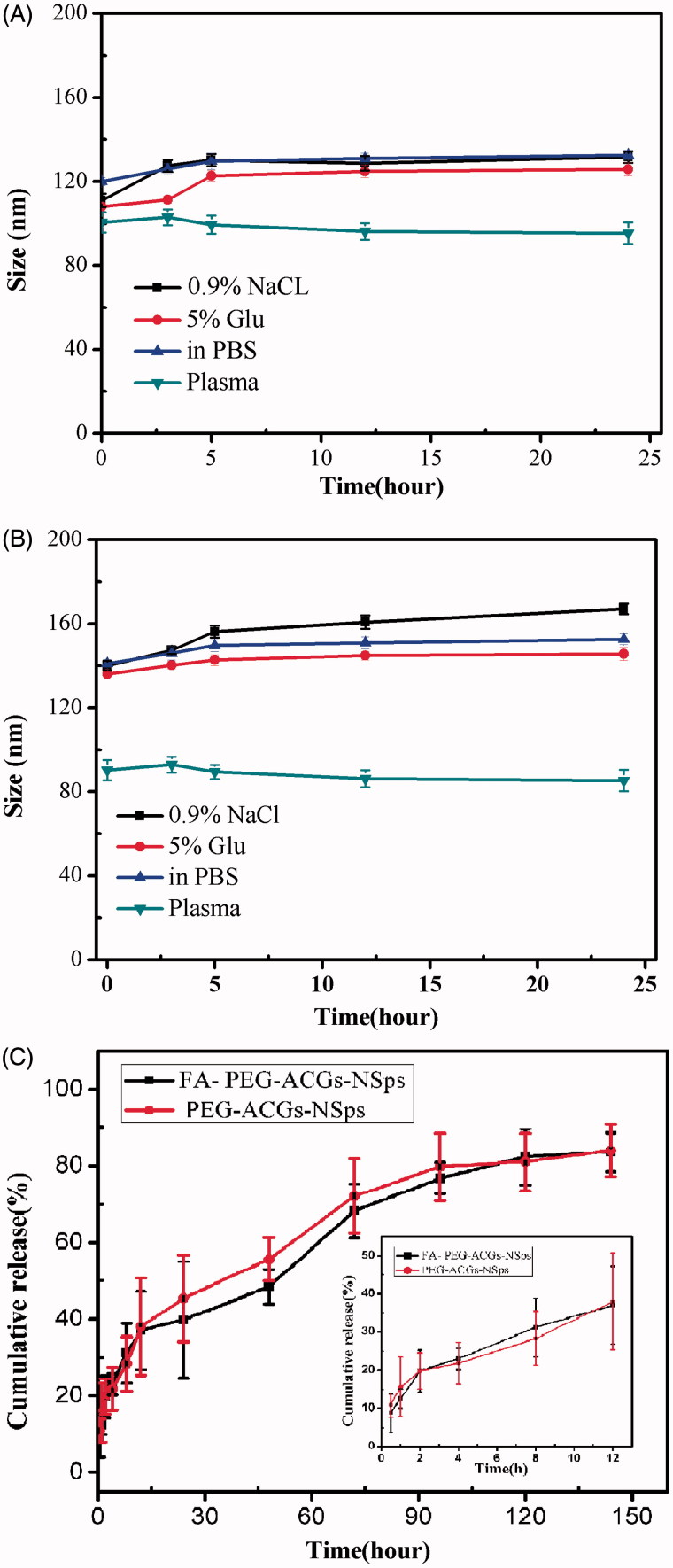
The stability and *in vitro* cumulative release profiles of ACGs-NSps. Particle size change of FA-PEG-ACGs-NSps (A) and PEG-ACGs-NSps (B) in 0.9% NaCl, 5% glucose, PBS, and plasma at 37 °C (*n* = 3, mean ± SD). (C) The *in vitro* cumulative release profiles of ACGs from FA-PEG-ACGs-NSps at 37 °C in pH 7.4 PBS. *Notes*: The amount of ACGs released from NSps was estimated by the reduction of quantity inside the dialysis bag with the HPLC method. All data are represented as mean ± SD (*n* = 3).

In order to know whether FA-PEG-ACGs-NPs and PEG-ACGs-NPs were suitable for intravenous administration, the size change of FA-PEG-ACGs-NPs and PEG-ACGs-NPs during the incubation in 0.9% NaCl, 5% glucose solution, PBS and plasma was investigated to understand their stability in these media. It was demonstrated that both FA-PEG-ACGs-NSps (Figure 2(A)) and PEG-ACGs-NSps (Figure 2(A)) were stable in normal saline, 5% glucose solution, PBS, and plasma, with little size enlargement after 24 h of incubation at 37 °C. This result demonstrated that the ACGs NSps can be adjusted into an isotonic solution and are suitable for *i.v.* administration. In the subsequent study, normal saline was chosen as the dispersion medium for FA-PEG-ACGs-NSps and PEG-ACGs-NSps for all the *in vivo* investigations, and PBS was chosen as the *in vitro* release medium.

### *In vitro* drug release study

The *in vitro* drug release from FA-PEG-ACGs-NSps was investigated using the dialysis bag diffusion method. PBS was selected as the *in vitro* release medium. Bullatacin, the most abundant compound in ACGs (Figure S1), was used as an indicator for the quantitative analysis of ACGs released from the nanoparticles. As seen in Figure 2(C), FA-PEG-ACGs-NPs and PEG-ACGs-NPs displayed quite similar cumulative dissolution profiles, suggesting FA-modification did not affect the drug release of ACGs-NSps. Both FA-PEG-ACGs-NPs and PEG-ACGs-NPs displayed a burst release within the first 2 h, with the cumulative drug release reaching about 20%, followed by a steady and sustained drug release phase with the cumulative drug release reaching up to 82.98% till 120 h. We also investigated the *in vitro* drug release of ACGs physical suspensions (dispersed directly in deionized water) under the same condition as a control. However, no drug release was detected during the whole process of trial (data not shown). This phenomenon may be because the solubility of ACGs is so poor that no drug was released from ACGs physical suspensions under the same condition.

The *in vitro* release of DiR from FA-PEG-ACGs/DiR-NSps and PEG-ACGs/DiR-NSps was also studied as a comparison. As seen in Figure S4, the cumulative release profile of DiR was similar to that of ACGs from both NSps, indicating that ACGs and DiR were released simultaneously and very slowly and thus DiR could be used to trace the biodistribution of FA-PEG-ACGs/DiR-NSps and PEG-ACGs/DiR-NSps in tumor-bearing model mice.

### *In vitro* cytotoxicity assay

**Figure 3.**
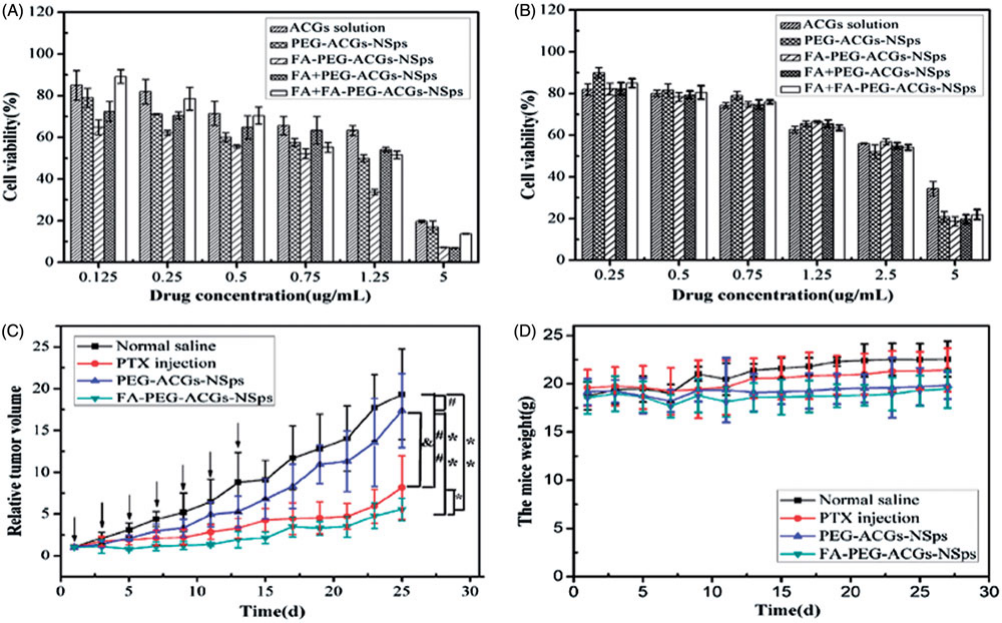
*In vitro* antiproliferative activity and *in vivo* antitumor experiments on HeLa tumor-bearing mice. (A) Cytotoxicity of ACGs solution, PEG-ACGs-NSps, FA-PEG-ACGs-NSps, FA + PEG-ACGs-NSps, and FA + FA-PEG-ACGs-NSps against HeLa cells (a) and A549 cells (b) for 24 h using MTT assay. All data are represented as mean ± SD (*n* = 6). (B) (a) The growth of relative tumor volume over time of PTX injections, PEG-ACGs-NSps, and FA-PEG-ACGs-NSps; (b) the average body weight change of mice along with time. All data are represented as mean ± SD. *p < .01, **p < .001 with FA-PEG-ACGs-NSps; &*p* < .01 with PEG-ACGs-NSps; #*p* < .01, ##*p* < .001 with normal saline. (C) The growth of relative tumor volume over time of PTX injections, PEG-ACGs-NSps, and FA-PEG-ACGs-NSps. (D) The average body weight change of mice along with time. All data are represented as mean ± SD. *p < 0.01, **p < 0.001 with FA-PEG-ACGs-NSps; &*p* < 0.01 with PEG-ACGs-NSps; #*p* < 0.01, ##*p* < 0.001with normal saline.

In this study, an MTT assay was used to investigate the cytotoxicity of FA-PEG-ACGs-NSps, PEG-ACGs-NSps, and ACGs free solution against FR-positive HeLa cells and FR-negative A549 cells. All the three ACGs formulations inhibited tumor cell growth in a dose-dependent manner. However, FA-PEG-ACGs-NSps showed stronger cytotoxicity against HeLa cells than PEG-ACGs-NSps and free ACGs (Figure 3(A)) at all of the tested concentrations. There was a statistical significance of HeLa cells viability between FA-PEG-ACGs-NSps and PEG-ACGs-NSps according to IC50 data (*p* < .05, Table 1).

**Table 1.** IC50 values of ACGs solution, PEG-ACGs-NSps, FA-PEG-ACGs-NSps, FA + PEG-ACGs-NSps, and FA + FA-PEG-ACGs-NSps against HeLa and A549 cell lines after incubation for 24 h.

	IC_50_ (μg/mL)		
Time	Groups	HeLa	A549
24 h	ACGs solution	1.308**	7.494
	PEG-ACGs-NSps	0.915*	7.285
	FA-PEG-ACGs-NSps	0.483	7.201
	FA + PEG-ACGs-NSps	0.936*	7.215
	FA + FA-PEG-ACGs-NSps	1.113*	7.254

Results are presented as mean ± SD; *n* = 6.

* *p* < .05.

** *p* < .01 vs. FA-PEG-ACGs-NSps.

In addition, the co-administration of FA showed no effect on the cytotoxicity of PEG-ACGs-NSps against HeLa cells (IC50, 0.915 μg/mL vs. 0.936 μg/mL, *p* > .05, Table 1), demonstrating that FA itself had neither cell growth inhibition against HeLa cells nor synergistic effect with ACGs. Differently, FA-PEG-ACGs-NSps displayed significantly higher cytotoxicity against HeLa cells than PEG-ACGs-NSps alone (IC50, 0.483 μg/mL vs. 0.915 μg/mL, *p* < .05, Table 1) or the mixture of FA and PEG-ACGs-NSps, suggesting that surface anchoring of FA molecules offered active targeting of PEG-ACGs-NSps to FA-positive HeLa cells. Correspondingly, the pretreatment of free FA reduced the cytotoxicity of FA-PEG-ACGs-NSps (IC50 value increasing from 0.483 μg/mL to 1.113 μg/mL, *p* < .05, Table 1), this proved that the original cytotoxicity enhancement was ascribed to the specific recognition of FR on HeLa cellular membrane by FA ligand of FA-PEG-ACGs-NSps, followed by FR-mediated endocytosis of ACGs NSp, and thus could be effectively blocked by the competitive inhibition of free FA pretreatment.

However, in case of FR-negative A549 cell line, due to very low FR expression on the cellular membrane, there was no difference in the cell viability (Figure 3(A,B)) or IC50 data (Table 1) among all of the ACGs formulations. Even ACGs nanoparticles failed to display higher cytotoxicity than ACGs/DMSO solution, this may be because A549 cell line was much less sensitive to ACGs than HeLa cell line, which was also evident from the comparison of their IC50 values (Table 1).

We also examined the cytotoxicity of free FA and blank nanoparticles prepared using DSPE-PEG-FA + SPC or DSPE-PEG + SPC, with nearly no cell cytotoxicity observed at all the tested concentrations against both HeLa and A549 cell lines (Figure S3). This proved that the drug carrier used in this study is safe and nontoxic.

### *In vivo* antitumor efficacy

The antitumor efficacy of FA-PEG-ACGs-NSps and PEG-ACGs-NSps was investigated in HeLa tumor-bearing mice using PTX injection as a positive control and normal saline as a negative control. As shown in Figure 3(C), the tumor volume in the saline control group increased rapidly and reached 4000 mm^3^ at the end of the experiment. PEG-ACGs-NSps displayed a significant tumor growth inhibition (*p* < .01, vs. normal saline), while FA-PEG-ACGs-NSps displayed an even better tumor growth inhibition (*p* < .01, vs. PEG-ACGs-NSps). As seen in Table 2, FA-PEG-ACGs-NSps (0.4 mg/kg) exhibited even superior TIR over PTX injection (8 mg/kg) (TIR, 76.45% vs. 53.23%, *p* < .05). However, PEG-ACGs-NSps (0.4 mg/kg) was not effective as PTX injection (TIR, 25.29% vs. 53.23%, *p* < .05). It was demonstrated that FA-PEG-ACGs-NSps and PTX injections showed persistent anti-tumor efficacy with no rebound phenomena after withdrawal of medication (Figure 3(C)). In general FA active targeting greatly enhanced the antitumor efficacy of ACGs NSps in FR-positive tumors compared to PEG-ACGs-NSps group.

**Table 2.** *In vivo* antitumor effects of different groups against HeLa tumors in mice.

Formulation	Tumor weight	Inhibition rate (%)
Normal saline	4.124 ± 0.237	NA
PTX injections (8 mg/kg)	1.927 ± 0.288**,&	53.23%
PEG-ACGs-NSps (0.4 mg/kg)	3.081 ± 0.252*	25.29%
FA-PEG-ACGs-NSps (0.4 mg/kg)	0.970 ± 0.057**^,^#,&&	76.45%

Tumor weight results are presented as mean ± SD, *n* = 8.

* *p* < .01.

** *p* < .001 vs. normal saline.

# *p* < .01 vs. PTX injection.

& *p* < .01.

&& *p* < .001 vs. PEG-ACGs-NSps.

We can see from Figure 3(D) that there was no significant average body weight change for mice in all groups during the whole process. On the whole, normal saline group showed relatively higher average body weight, followed by PTX group. Even for FA-PEG-ACGs-NSps group, no average body weight reduction was observed, indicating good bio-safety concurrent with superior antitumor efficacy. All the above findings suggested that FA modified PEGylation active targeting ACGs-NSPs were promising in cancer treatment.

### *In vivo* biodistribution

As none of ACGs effective components had ultraviolet or fluorescent absorption able to provide ACGs with a sensitive *in vivo* quantitative analysis method (Hong et al., 2016), DiR was incorporated in ACGs-NSps to estimate the *in vivo* distribution behavior of ACGs in HeLa tumor-bearing mice. Whatever for FA-PEG-ACGs/DiR-NSps or for PEG-ACGs/DiR-NSps, ACGs and DiR were slow but nearly synchronously released in the *in vitro* experiment (Figure S4). This meant that DiR could be used to trace the *in vivo* behaviors of ACGs-NSps under particular circumstances.

**Figure 4.**
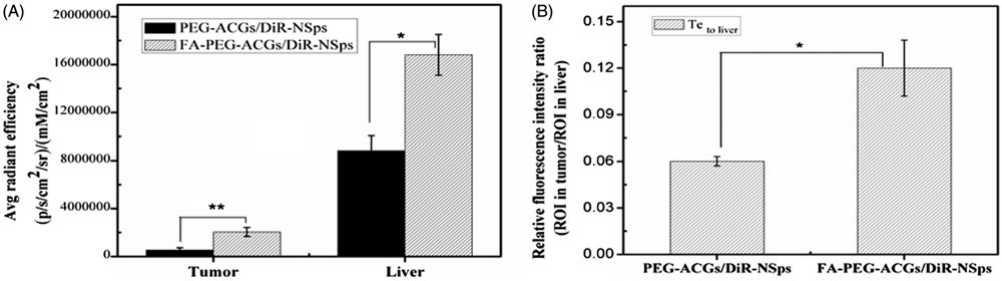
The *in vivo* biodistribution of PEG-ACGs/DiR-NSps, and FA-PEG-ACGs/DiR – NSps in HeLa tumor-bearing mice. (A) Average fluorescence intensity in tumors of two groups at the end of the experiment. (B) The ratio of the fluorescence intensity in tumor to the fluorescence intensity in liver (Te to liver) of two groups at the end of the experiment. All data are represented as mean ± SD. *p < .05, **p < .01 with FA-ACGs-NSps.

Figure 4(A) shows the fluorescence intensity in tumor and liver of mice. Most fluorescence was found in liver, which was consistent with previous report (Weitman et al., 1992; Solanky et al., 2010; Xu et al., 2017). The *i.v.* administrated NSps may be recognized as foreign objects and then be partially uptaken by the mononuclear phagocyte system which abounded in liver and spleen (Weitman et al., 1992). The FA-PEG-ACGs/DiR-NSps group displayed much more fluorescence in the tumor than PEG-ACGs/DiR-NSps group (*p* < .01, Figure 4(A)), probably due to the specific interaction between the FA ligand of FA-PEG-ACGs/DiR-NSps and FR of HeLa tumor cells successfully retaining FA-PEG-ACGs/DiR-NSps in tumor tissues from the circulation, thus enhancing the cellular uptake.

In order to evaluate the targeting efficiency (Te) of drug delivery systems, the ratio of the fluorescence intensity in tumor to the fluorescence intensity in liver was calculated and used as an index here (Christoph et al., 2013). Figure 4(B) shows that FA-PEG-ACGs/DiR-NSps had a better Te than PEG-ACGs/DiR-NSps (*p* *<* .05, Figure 4(B)). It proved that FA modification did provide ACGs-NSps with good active tumor targeting ability in FR-positive tumors.

## Conclusions

ACGs are a series of active components isolated from natural resources with strong toxicity against various tumor cells. However, further clinic application has been limited due to the poor solubility and serious side effect. Using DSPE-PEG-FA and SPC in combination as stabilizers, we successfully prepared FA-targeting and PEG modified ACGs-NSps with a small size and good stability. *In vitro* cytotoxicity experiments proved that FA-PEG-ACGs-NSps could be specifically recognized and enter FR-positive cells via FR-mediated endocytosis. The *in vivo* experiments demonstrated that FA-PEG-ACGs-NSps could efficiently lead encapsulated drugs to the tumor site and meanwhile greatly enhanced the *in vivo* antitumor efficacy after intravenous administration. Our present and previous studies on active targeted ACGs-NSps all suggested that nanotechnology in combination with active tumor targeting drug delivery systems is very helpful to expand the therapeutical window of ACGs by enhancing the antitumor efficacy and the consequent dose reduction. These findings may provide new ideas and methods to the application of ACGs in clinical cancer treatment, although there is still much work to do.

## Supplementary Material

IDRD_Wang_et_al_Supplemental_Content.docx

## References

[CIT0001] Aditya NP, Yang H, Kim S, Ko S. (2015). Fabrication of amorphous curcumin nanosuspensions using β-lactoglobulin to enhance solubility, stability, and bioavailability. Colloids Surf B Biointerfaces 127:114–21. 25660094 10.1016/j.colsurfb.2015.01.027

[CIT0002] Ahammadsahib KI, Hollingworth RM, McGovren JP, et al (1993). Mode of action of bullatacin: a potent antitumor and pesticidal annonaceous acetogenin. Life Sci 53:1113–20. 8371627 10.1016/0024-3205(93)90547-g

[CIT0003] Bermejo A, Figadere B, Zafrapolo MC, et al (2005). Acetogenins from Annonaceae: recent progress in isolation, synthesis and mechanisms of action. Nat Prod Rep 36:269–303. 10.1039/b500186m 15806200

[CIT0004] Bomfim LM, Menezes LRA, Rodrigues ACBC, et al (2016). Antitumour activity of the microencapsulation of *Annona vepretorum* essential oil. Basic Clin Pharmacol Toxicol 118:208–13. 26348780 10.1111/bcpt.12488

[CIT0005] Bueno R, Appasani K, Mercer H, et al (2001). The alpha folate receptor is highly activated in malignant pleural mesothelioma. J Thor Cardiovasc Surg 121:225–33. 10.1067/mtc.2001.111176 11174727

[CIT0006] Chen Y, Chen JW, Li X. (2011). Cytotoxic bistetrahydrofuran annonaceous acetogenins from the seeds of *Annona squamosa*. J Nat Prod 74:2477–81. 22011319 10.1021/np200708q

[CIT0007] Chen Y, Chen JW, Liu SJ, et al (2012). Determination of bullatacin in rat plasma by liquid chromatography–mass spectrometry. J Chromatogr B Anal Technol Biomed Life Sci 897:94–7. 10.1016/j.jchromb.2012.03.044 22534655

[CIT0008] Christoph DC, Asuncion BR, Hassan B, et al (2013). Significance of folate receptor alpha and thymidylate synthase protein expression in patients with non-small-cell lung cancer treated with pemetrexed. J Thor Oncol 8:19–30. 10.1097/JTO.0b013e31827628ff PMC3645936 23242435

[CIT0009] Dang YJ, Feng HZ, Zhang L, et al (2012). In situ absorption in rat intestinal tract of solid dispersion of annonaceous acetogenins. Gastroenterol Res Pract 2012:879676. 22536222 10.1155/2012/879676 PMC3303619

[CIT0010] Degli EM, Ghelli A, Ratta M, et al (1994). Natural substances (acetogenins) from the family Annonaceae are powerful inhibitors of mitochondrial NADH dehydrogenase (Complex I). Biochem J 301:161. 8037664 10.1042/bj3010161 PMC1137156

[CIT0011] Duval RA, Lewin G, Peris E, et al (2006). Heterocyclic analogues of squamocin as inhibitors of mitochondrial complex I. On the role of the terminal lactone of annonaceous acetogenins. Biochemistry 45:2721–8. 16489765 10.1021/bi051261u

[CIT0012] Formagio ASN, Vieira MC, Volobuff CRF, et al (2015). In vitro biological screening of the anticholinesterase and antiproliferative activities of medicinal plants belonging to Annonaceae. Braz J Med Biol Res 48:308–15. 25714885 10.1590/1414-431X20144127 PMC4418360

[CIT0013] Hong J, Li Y, Xiao Y, et al (2016). Annonaceous acetogenins nanosuspensions stabilized by PCL-PEG block polymer: significantly improved antitumor efficacy. IJN 11:3239. 27486323 10.2147/IJN.S108143 PMC4957684

[CIT0014] Jain KK. (2005). Nanotechnology-based drug delivery for cancer. Technol Cancer Res Treat 4:407–16. 16029059 10.1177/153303460500400408

[CIT0015] Jain RK, Dan GD, Batchelor TT, et al (2008). Normalization of tumor vasculature and microenvironment. New York: Springer.

[CIT0016] Kalli KR, Oberg AL, Keeney GL, et al (2008). Folate receptor alpha as a tumor target in epithelial ovarian cancer. Gynecol Oncol 108:619. 18222534 10.1016/j.ygyno.2007.11.020 PMC2707764

[CIT0017] Kennedy MD, Low PS. (2011). Folate targeted enhanced tumor and folate receptor positive tissue optical imaging technology. US.

[CIT0018] Leamon CP, Low PS. (1991). Delivery of macromolecules into living cells: a method that exploits folate receptor endocytosis. Proc Natl Acad Sci USA 88:5572–6. 2062838 10.1073/pnas.88.13.5572 PMC51919

[CIT0019] Leamon CP, Reddy JA. (2004). Folate-targeted chemotherapy. Adv Drug Deliv Rev 56:1127. 15094211 10.1016/j.addr.2004.01.008

[CIT0020] Lu Y, Sega E, Low PS. (2005). Folate receptor-targeted immunotherapy: induction of humoral and cellular immunity against hapten-decorated cancer cells. Int J Cancer 116:710–9. 15828051 10.1002/ijc.21126

[CIT0021] Matherly LH, Goldman DI. (2003). Membrane transport of folates. Vitam Horm 66:403. 12852262 10.1016/s0083-6729(03)01012-4

[CIT0022] Motoyama T, Yabunaka H, Miyoshi H. (2002). Essential structural factors of acetogenins, potent inhibitors of mitochondrial complex I. Bioorg Med Chem Lett 33:2089–92. 10.1016/s0960-894x(02)00374-8 12127510

[CIT0023] Pan XP, Yu DQ. (1997). Studies on new cytotoxic annonaceous acetogenins from Uvaria grandiflora and absolute configurations. Acta Pharm Sin 32:286. 11499032

[CIT0024] Pieme CA, Kumar SG, Dongmo MS, et al (2014). Antiproliferative activity and induction of apoptosis by *Annona muricata* (Annonaceae) extract on human cancer cells. BMC Complement Altern Med 14:516. 25539720 10.1186/1472-6882-14-516 PMC4324658

[CIT0025] Reddy JA, Westrick E, Vlahov I, et al (2006). Folate receptor specific anti-tumor activity of folate–mitomycin conjugates. Cancer Chemother Pharmacol 58:229–36. 16331500 10.1007/s00280-005-0151-z

[CIT0026] Rupprecht JK, Hui YH, McLaughlin JL. (1990). Annonaceous acetogenins: a review. J Nat Prod 53:237. 2199608 10.1021/np50068a001

[CIT0027] Solanky N, Requena JA, D'Souza SW, et al (2010). Expression of folate transporters in human placenta and implications for homocysteine metabolism. Placenta 31:134. 20036773 10.1016/j.placenta.2009.11.017

[CIT0028] Sun S, Liu J, Kadouh H, et al (2014). Three new anti-proliferative annonaceous acetogenins with mono-tetrahydrofuran ring from graviola fruit (*Annona muricata*). Bioorg Med Chem Lett 24:2773. 24780120 10.1016/j.bmcl.2014.03.099

[CIT0029] Sun S, Liu J, Zhou N, et al (2015). Isolation of three new annonaceous acetogenins from Graviola fruit (*Annona muricata*) and their anti-proliferation on human prostate cancer cell PC-3. Bioorg Med Chem Lett 26:4382–5. 27499453 10.1016/j.bmcl.2015.06.038

[CIT0030] Torchilin VP. (2007). Micellar nanocarriers: pharmaceutical perspectives. Pharm Res 24:1. 17109211 10.1007/s11095-006-9132-0

[CIT0031] Tormo JR, Estornell E. (2000). New evidence for the multiplicity of ubiquinone- and inhibitor-binding sites in the mitochondrial complex I. Arch Biochem Biophys 381:241. 11032411 10.1006/abbi.2000.1969

[CIT0032] Tormo JR, Gallardo T, Aragón R, et al (1999). Specific interactions of monotetrahydrofuranic annonaceous acetogenins as inhibitors of mitochondrial complex I. Chem-Biol Interact 122:171–83. 10682937 10.1016/s0009-2797(99)00120-9

[CIT0033] Weitman SD, Lark RH, Coney LR, et al (1992). Distribution of the folate receptor GP38 in normal and malignant cell lines and tissues. Cancer Res 52:3396–401. 1596899

[CIT0034] Xu X, Saw PE, Tao W, et al (2017). ROS‐responsive polyprodrug nanoparticles for triggered drug delivery and effective cancer therapy. Adv Mater 29:1700141. 10.1002/adma.201700141 PMC5681219 28681981

[CIT0035] Yang H, Zhang N, Li X, et al (2009). Structure-activity relationships of diverse annonaceous acetogenins against human tumor cells. Bioorg Med Chem Lett 19:2199–202. 19285863 10.1016/j.bmcl.2009.02.105

[CIT0036] Yang L, Hong J, Di J, et al (2017). 10-Hydroxycamptothecin (HCPT) nanosuspensions stabilized by mPEG1000-HCPT conjugate: high stabilizing efficiency and improved antitumor efficacy. IJN 12:3681. 28553107 10.2147/IJN.S134005 PMC5439984

[CIT0037] Zafra-Polo MC, Gonzalez MC, Estornell E, et al (2010). ChemInform abstract: acetogenins from annonaceae, inhibitors of mitochondrial complex I. Cheminform 27:253–71. 10.1016/0031-9422(95)00836-5 8688168

