# Supplementary material for: Folate-targeting annonaceous acetogenins nanosuspensions: significantly enhanced antitumor efficacy in HeLa tumor-bearing mice
Source: Drug Deliv. 2018 Apr 2;25(1):880–7. doi: 10.1080/10717544.2018.1455761 (PMC6058653; doi:10.1080/10717544.2018.1455761)
Supplement: IDRD_Wang_et_al_Supplemental_Content.docx [file IDRD_A_1455761_SM8822.docx]

Supplementary Materials

Table S1. Content percentage of the five major components (%) for five batches of ACGs

| Batch | Content percentage of the 5 major components (%) | | | | | Total  content (%) |
| --- | --- | --- | --- | --- | --- | --- |
|  | **K20** | **GK23** | **K437** | **K19** | **K16** |  |
| 091  092  093  094  095 | 0.142 | 1.308 | 8.929 | 43.124 | 12.145 | 65.695 |
|  | 3.214 | 0.854 | 8.443 | 39.316 | 10.813 | 62.640 |
|  | 3.185 | 1.209 | 7.409 | 36.375 | 9.752 | 57.930 |
|  | 3.470 | 1.593 | 7.729 | 37.387 | 8.984 | 59.163 |
|  | 3.723 | 1.798 | 7.480 | 37.635 | 9.172 | 59.808 |


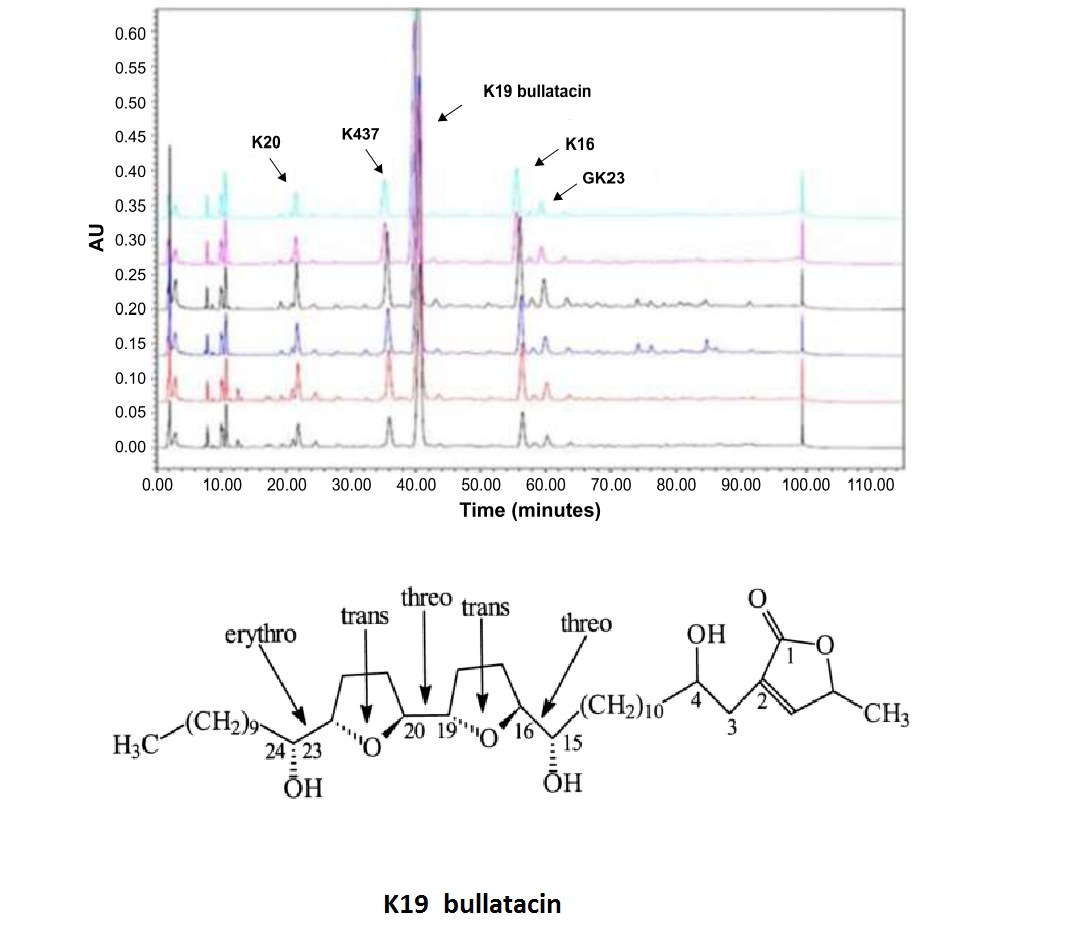


Figure S1. HPLC fingerprint chromatograms determined at 210 nm for five batches of total annonaceous acetogenins and the chemical structure of K19 (bullatacin).

Table S2. The size and zeta potential of the resultant ACGs-NSps with or without SPC with the final drug concentration of 1mg/mL

| No | ACGs(mg) | DSPE-PEG(mg) | DSPE-PEG-FA(mg) | SPC(mg) | Size  (nm) | PDI | Zeta  (mV) |
| --- | --- | --- | --- | --- | --- | --- | --- |
| 1 | 6 | 3 | 0 | 0 | 144.3 | 0.135 | -21.9 |
| 2 | 6 | 3 | 0 | 3 | 139.0 | 0.209 | -30.1 |
| 3 | 6 | 0 | 3 | 0 | 145.5 | 0.281 | -25.4 |
| 4 | 6 | 0 | 3 | 3 | 119.7 | 0.200 | -23.0 |


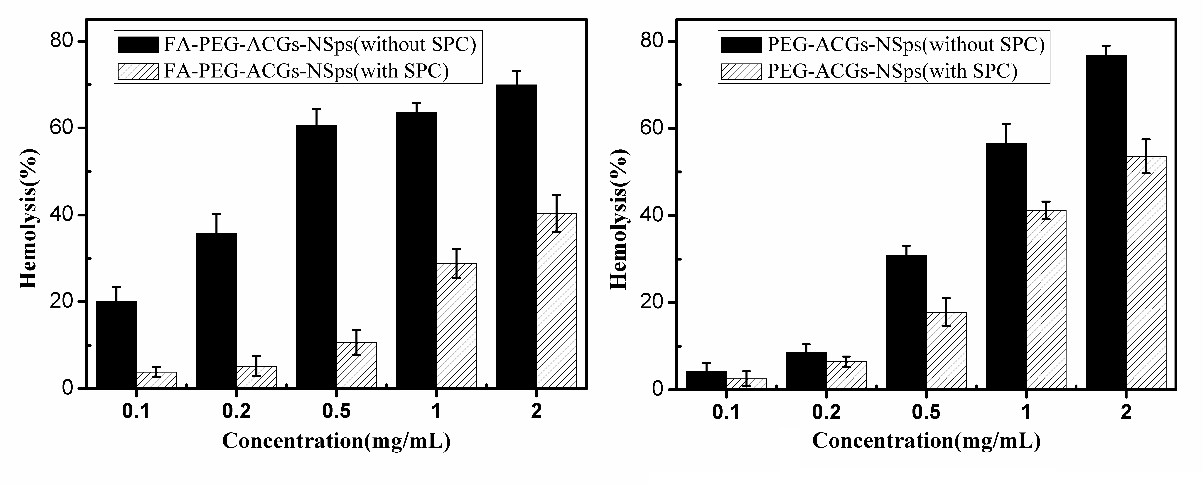


Figure S2. Hemolysis percentage of the ACGs-NSps prepared with or without SPC in the formulation. All data are represented as mean ± SD (n=3)

Table S3. Various formulations and the size and zeta potential of the resultant FA-PEG-ACGs-NSps with the final drug concentration of 1mg/mL

| No | ACGs(mg) | DSPE-PEG-FA(mg) | SPC(mg) | DSPE-PEG-FA  /SPC | Size  (nm) | PDI | Zeta  (mV) |
| --- | --- | --- | --- | --- | --- | --- | --- |
| 1 | 6 | 5 | 1 | 5:1 | 161.5 | 0.117 | -37.9 |
| 2 | 6 | 4.5 | 1.5 | 3:1 | 174.5 | 0.157 | -20.8 |
| 3 | 6 | 3 | 3 | 1:1 | 119.7 | 0.200 | -23.0 |
| 4 | 6 | 1.5 | 4.5 | 1:3 | 146.0 | 0.160 | -23.1 |
| 5 | 6 | 1 | 5 | 1:5 | 152.2 | 0.175 | -23.8 |

Table S4. The size and zeta potential of FA-PEG-ACGs/DiR-NSps and PEG-ACGs/DiR-NSps with the final drug concentration of 1mg/mL

| Formulations | Size(nm) | PDI | Zeta(mV) |
| --- | --- | --- | --- |
| FA-PEG-ACGs/DiR-NSps | 131.0 | 0.183 | -30.1 |
| PEG-ACGs/DiR-NSps | 132.5 | 0.207 | -36.1 |


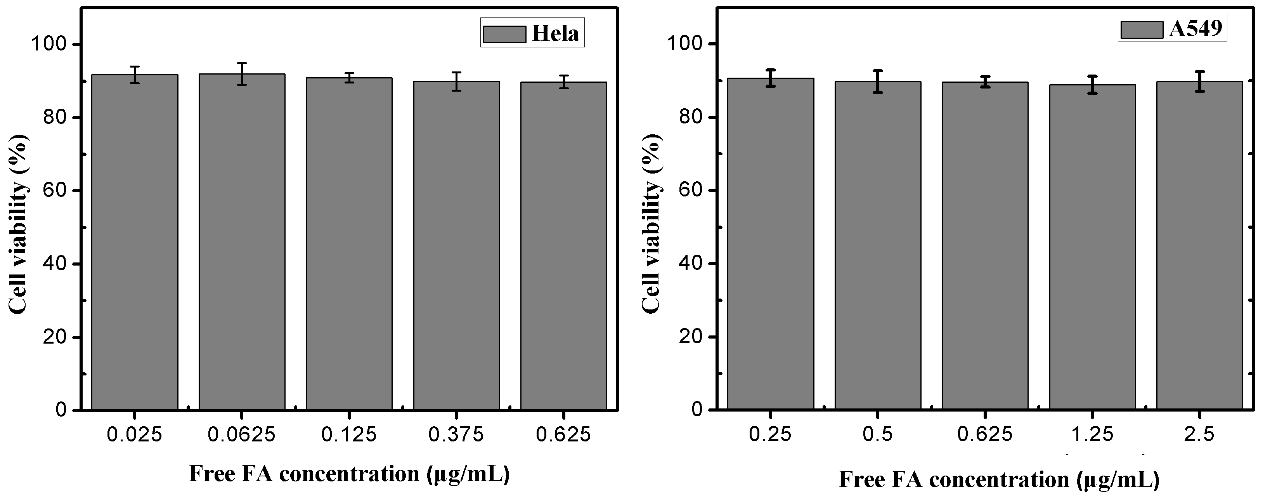


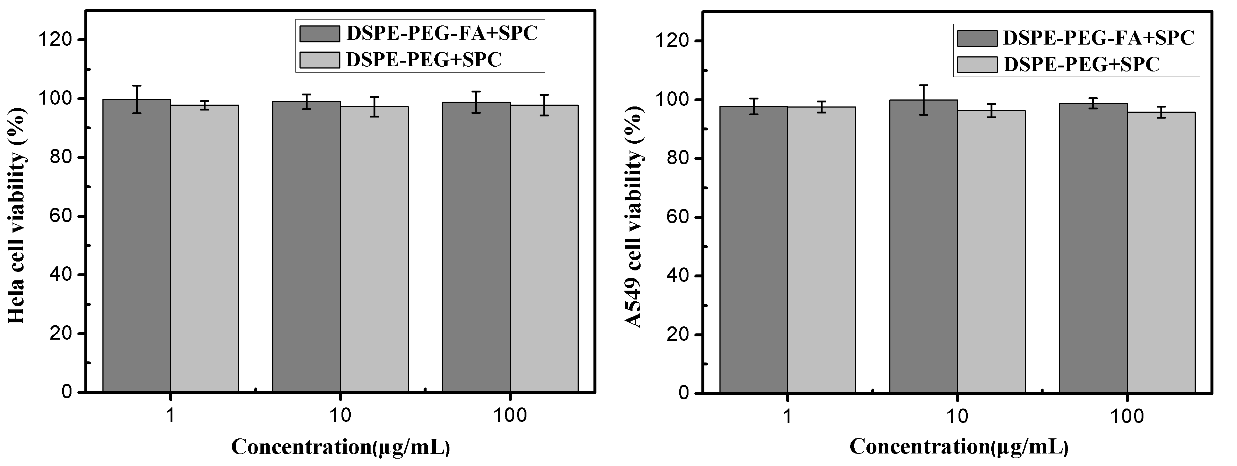


Figure S3. Cytotoxicity of free FA and blank NSps prepared by DSPE-PEG-FA or DSPE-PEG against Hela or A549 cells for 24 hours.

Note: all data are represented as mean ± SD (n=6).


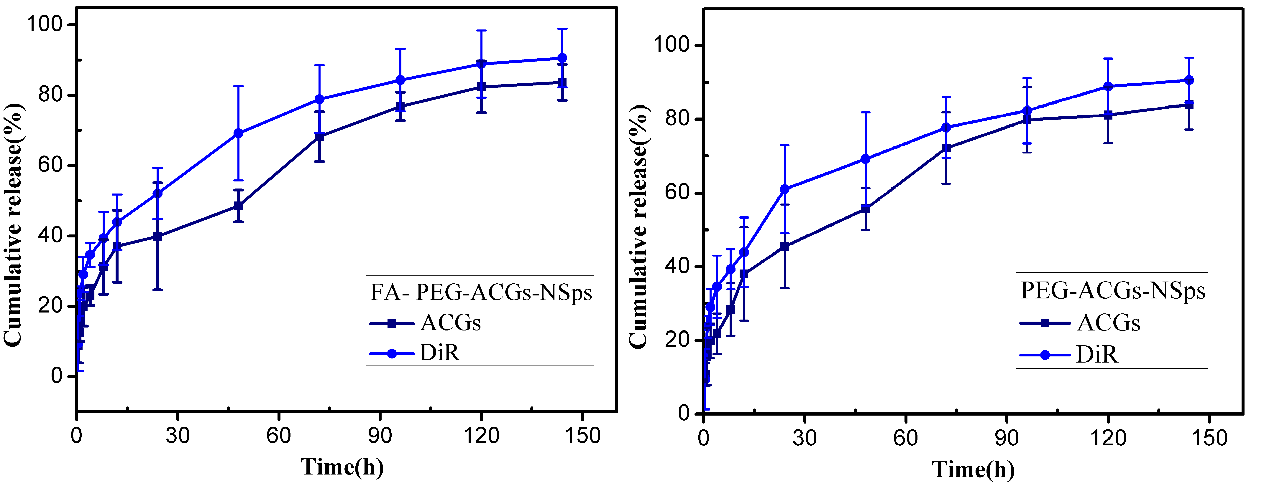


Figure S4. In vitro cumulative release profiles of ACGs and DIR from FA-PEG-ACGs/DiR-NSps and PEG-ACGs/DiR-NSps in pH 7.4 PBS at 37°C.

Notes: The amount of ACGs and DiR released from NSps was estimated by the reduction of quantity inside the dialysis bag with the HPLC method. All data are represented as mean ±SD (n=3).
